# Magnetic liposomes for colorectal cancer cells therapy by high-frequency magnetic field treatment

**DOI:** 10.1186/1556-276X-9-497

**Published:** 2014-09-15

**Authors:** Andri Hardiansyah, Li-Ying Huang, Ming-Chien Yang, Ting-Yu Liu, Sung-Chen Tsai, Chih-Yung Yang, Chih-Yu Kuo, Tzu-Yi Chan, Hui-Ming Zou, Wei-Nan Lian, Chi-Hung Lin

**Affiliations:** 1 Department of Materials Science and Engineering, National Taiwan University of Science and Technology, Taipei, 106, Taiwan; 2 Department of Materials Engineering, Ming Chi University of Technology, New Taipei City, 24301, Taiwan; 3 Institute of Microbiology and Immunology, School of Life Science, National Yang-Ming University, Taipei, 11221, Taiwan; 4 Institute of Polymer Science and Engineering, National Taiwan University, Taipei, 106, Taiwan

**Keywords:** Liposomes, Magnetic nanoparticle, Colorectal cancer, High-frequency magnetic field, Drug controlled release

## Abstract

In this study, we developed the cancer treatment through the combination of chemotherapy and thermotherapy using doxorubicin-loaded magnetic liposomes. The citric acid-coated magnetic nanoparticles (CAMNP, *ca.* 10 nm) and doxorubicin were encapsulated into the liposome (HSPC/DSPE/cholesterol = 12.5:1:8.25) by rotary evaporation and ultrasonication process. The resultant magnetic liposomes (*ca.* 90 to 130 nm) were subject to characterization including transmission electron microscopy (TEM), dynamic light scattering (DLS), X-ray diffraction (XRD), zeta potential, Fourier transform infrared (FTIR) spectrophotometer, and fluorescence microscope. *In vitro* cytotoxicity of the drug carrier platform was investigated through 3-(4,5-dimethylthiazol-2-yl)-2,5-diphenyltetrazolium bromide (MTT) assay using L-929 cells, as the mammalian cell model. *In vitro* cytotoxicity and hyperthermia (inductive heating) studies were evaluated against colorectal cancer (CT-26 cells) with high-frequency magnetic field (HFMF) exposure. MTT assay revealed that these drug carriers exhibited no cytotoxicity against L-929 cells, suggesting excellent biocompatibility. When the magnetic liposomes with 1 μM doxorubicin was used to treat CT-26 cells in combination with HFMF exposure, approximately 56% cells were killed and found to be more effective than either hyperthermia or chemotherapy treatment individually. Therefore, these results show that the synergistic effects between chemotherapy (drug-controlled release) and hyperthermia increase the capability to kill cancer cells.

## Background

Development of smart materials that could response to the environmental stimuli is gaining importance over the past decade in the drug delivery system [1]. Remote control drug carrier behavior has been regarded as a function that could enhance the efficacy and efficiency of drug delivery to the target sites [2,3]. Among several drug carrier candidates, liposomes exhibit a number of excellences. Liposomes are synthetic lipid bilayer with enclosed structure up to several hundred nanometers in diameter. It is highly biocompatible and biodegradable and can encapsulate both hydrophilic and hydrophobic pharmaceutical agents and protect them from the inactivating effect of external condition. Moreover, liposomes provide a unique characteristic to deliver pharmaceuticals into cells or even inside individual compartments [4-7]. Recently, researchers have developed novel liposomes to provide a smart treatment in human body which can undergo the releasing of encapsulated contents as the response to the environmental stimuli like temperature [2], pH [8], light [9], ultrasound [10,11], magnetic field [12,13], and so on. These specific environment stimuli are used as the driving force for triggered drug release based on the interaction between the stimuli and liposomes. Among the aforementioned stimuli, magnetic-triggered system has become one of the most potential strategies as the release and targeting stimuli.

Recently, iron oxide (Fe_3_O_4_)-based magnetic nanoparticles (MNP) has aroused great interest in magnetic-based releasing system in biomedical applications due to their physical properties and biocompatible nature [14,15]. MNP for biomedical applications should be hydrophilic and stable in water [16]. Aqueous colloidal dispersions of MNP hold great potential for use in a variety of novel and existing bioprocesses because of the compatibility of the aqueous medium with biosystem [17,18]. A number of surfactants or compounds have been reported as a stabilizing agent of MNP. Among of them, citric acid (CA) is frequently used to obtain an aqueous stable dispersion of MNP [17,19-23]. Targeting drug delivery system can be divided into two general categories: passive and active. MNP provided the main advantages of both types of targeting: as with passive targeting, modification of the nanoparticle surface is not necessary, and like active targeting, they can be directed to the site of interest [2]. This is due to the fact that MNP respond strongly to the magnetic fields, and magnetic fields can penetrate human tissue without impediment [24]. Furthermore, MNP accumulated in a tumor can act as hyperthermia-inducing agents, using the exothermic properties derived from hysteresis and/or Néel relaxation losses by application of high-frequency magnetic field (HFMF) to raise the local temperature around the cells [16,23,25]. Moreover, MNP have been used as a part of integrating system with liposomes termed as magnetic liposomes or magnetoliposomes [24-26].

HFMF is one of the most promising external stimulus because it is less invasive than other methods and has a high permeability in relation to the human body and can be operated remotely. HFMF can depress the drug-drug carrier interaction and accelerate diffusion [16]. Drug release rate is significantly enhanced in the presence of a magnetic field because of the pulsatile mechanical deformation that generates compressive and tensile stresses. Moreover, HFMF-triggered drug delivery system utilized the collapse or the volume transition of drug carriers then induce the drug release [27]. Previous study confirmed that the combination of dual-functional (magnetic and thermal) drug carriers upon the exposure to the HFMF upon a short time could lead to heating effect and a rapid release, hence could enhance the wide application for biomedical applications [28].

In the present work, we reported the preparation and evaluation of doxorubicin-loaded magnetic liposomes for colorectal cancer cells (CT-26 cells) treatment. The resultant of doxorubicin-loaded magnetic liposomes were subject to characterization including transmission electron microscopy (TEM), dynamic light scattering (DLS), X-ray diffraction (XRD), zeta potential, Fourier transform infrared (FTIR) spectrophotometer, and fluorescence microscopy. Biocompatibility was evaluated with mammalian cells (L-929 fibroblast). Further, the efficacy of this drug carrier against CT-26 cells with HFMF exposure was conducted to define the efficacy of this treatment.

## Methods

### Synthesis of aqueous stable CAMNP

The coprecipitation method was used for the synthesis of citric acid-coated magnetic nanoparticles as the method described previously with minor modification [14,18,19]. Briefly, FeCl_3_.6H_2_O and FeCl_2_.4H_2_O (molar ratio of 2:1) were mixed using double-distilled water and stirred at 1,000 rpm in a three-necked flask. The temperature of the solution was increased to 80°C in N_2_ atmosphere and kept for 30 min. Further, 20 mL of ammonia solution was added while mixing. The mixture was allowed to complete the magnetite formation for 30 min; afterwards, 0.5 g/mL CA was added, and the reaction temperature was increased to 90°C and kept for 1 h with continuous stirring. The black precipitates were harvested by cooling the reaction mixture to the room temperature, and then the particles were allowed to settle down with the help of a magnet. Further, these magnetic nanoparticles were washed carefully using double-distilled water. The products were dried under vacuum at room temperature and termed as citric acid-coated magnetic nanoparticles (CAMNP).

### Preparation of liposomes and DOX-loaded magnetic liposomes

Liposomes composed of fully hydrogenated soy phosphatidylcholine (HSPC)/1,2-distearoyl-*sn*-glycero-3-phosphoethanolamine (DSPE)/cholesterol at mole ratios of 12.5:1:8.25 were prepared via conventional thin film hydration technique as method described previously with minor modification [29,30]. Briefly HSPC, DSPE, and cholesterol were dissolved in chloroform and ethanol (3:1 *v*/*v*). The mixture was transferred into a 300-mL round bottom flask and placed to rotary evaporation system (N-1200 series, Eyela®, Tokyo Rikakikai Co., Ltd., Tokyo, Japan) for the elimination of any traces and residual of organic solvents. Further, a thin dry lipid film would form on the wall of the round bottom flask. This thin dry lipid film was kept in rotary evaporation system for 6 h to ensure complete removal of organic solvents. Hydration process of the dry lipid film was accomplished by adding the phosphate buffer solution (PBS) of pH 7.4, which resulted in liposome suspension. In addition, CAMNP and doxorubicin (DOX)/CAMNP were introduced in the hydration process to obtain magnetic liposomes and DOX-loaded magnetic liposomes, respectively. This suspension was subjected to a bath-type sonicator (Ultrasonic Cleaner, Kudos, Shanghai, China) for harvesting the liposomes. Afterward, the liposomes were homogenized using ultrasonicator (Probe-type sonicator, VCX 750, Vibra-Cell™, SONICS®, Sonics and Materials, Inc., Newton, CT, USA) at 24 W for 10 min. The suspension was then centrifuged at 1,000 × *g* for 15 min, which precipitated unincorporated magnetic particles at the bottom of the tube and retained the drug-containing magnetic liposomes in the supernatant [13]. Further, the suspension was dialyzed to remove unincorporated DOX [31]. Then, the suspension was extruded through a 0.22-μm filter for sterilization and to reduce the size. Furthermore, the resulting suspensions of liposomes were then preserved at 4°C prior to characterizations.

### Characterization

Particle size was measured using a DLS spectrophotometry (Horiba Instrument, Horiba, Kyoto, Japan) with helium-neon laser with wavelength of 633 nm, scattering angle of 90°, and refractive index of 1.33 at 25°C. The zeta potential was determined through electrophoretic mobility measurement (Horiba Instrument) with the following specifications: a dispersion medium viscosity of 0.894 mPa.s, a refractive index of 1.33, and temperature of 25°C. The structure and morphology of liposomes and doxorubicin liposomes were examined using transmission electron microscope (TEM-7650, Hitachi, Chiyoda-ku, Japan) at an acceleration voltage of 75 kV. Phosphotungstic acid (PTA) 1% *w*/*v* was used as the staining agent. For CAMNP and DOX-loaded magnetic liposomes, TEM was conducted without using PTA. XRD was conducted using a D_2_ Phaser BRUKER X-ray powder diffractometer (Bruker AXS, Inc., Madison, WI, USA) to evaluate the crystallographic characteristics by scanning dried powder in the 2*θ* range of 20° to 70° with Cu K_α-1_ (1.5406 Å) radiation. The infrared spectra were recorded using a FTIR-460 PLUS (Jasco Co. Ltd., Tokyo, Japan) to determine the major characteristic functional groups. Briefly, the sample was mixed with KBr pellet, and the mixtures were pressed into a pellet form prior to characterization. The incorporation of DOX into the drug carrier was visualized using fluorescence microscope (Olympus, Shinjuku-ku, Japan) equipped with a × 40 objective. Fluorescence images were obtained with a fluorescence microscope (Olympus) at wavelengths of 490 (excitation) and 590 nm (emission). The amount of DOX encapsulated into the liposomes or DOX-loaded magnetic liposomes was determined by a fluorescence spectrophotometer at wavelength of 490 nm for excitation and 590 nm for emission, after lysis of liposomes with a sufficient amount of acetonitrile [13]. The encapsulation efficiency (EE) was calculated by the amount of drug encapsulated/initial drug loading × 100%.

### Hyperthermia experiment

Hyperthermia experiment was performed as the method reported previously with minor modification [32]. Adiabatic condition is assumed, where the initial temperature of the sample must be equal to the temperature of the surrounding medium [33]. Therefore, the temperature of the surrounding medium was kept constant using a heated chamber at 37°C then the initial temperature was 37°C for all of the measurements. Briefly, CAMNP and or DOX-loaded magnetic liposomes suspension were subjected to the center of copper coil in the HFMF apparatus for 10 min. The change of the temperature was recorded every 2 min by an alcohol thermometer. Each experiment was performed three times.

### *In vitro* drug release test

*In vitro* drug release test was conducted to evaluate the release behavior of DOX from drug carriers during the exposure of HFMF. Before conducting drug release test, the system was first purified using centrifugation in order to remove the unincorporated magnetic nanoparticles [25]. Briefly, doxorubicin liposomes and DOX-loaded magnetic liposomes were placed into glass tube which contained the release solution (pH 4) and placed under HFMF for 10 min. About 1 mL of drug was taken from the test tube during the test for 2 min first and continued 2 min until 10 min during HFMF exposure. The quantity of DOX was quantified using UV-visible spectrophotometer at wavelength of 480 nm.

The drug release percent was evaluated by applying Equation 1:

(1) $$\mathrm{Cumulative}\;\mathrm{release}\;\left(\%\right)\;=\;R_{t}/D\;\times \;100\%$$

where *D* and *R*_*t*_ represent the initial amount of drug loaded and the cumulative amount of drug released at time *t*, respectively.

### Cell culture

Mouse fibroblasts (L-929 cells) were obtained from ATCC CRL-1503TM. Dulbecco's modified Eagle's medium high glucose (DMEM), trypsin, dimethylsulfoxide (DMSO), trypan blue, and 3-(4,5-dimethylthiazo-2-yl)-2,5-diphenyl tetrazolium bromide (MTT) powder were purchased from Sigma Aldrich, St. Louis, MO, USA. Fetal bovine serum (FBS) was purchased from BD Biosciences, San Jose, CA, USA. Fibroblasts cells (L929) were cultured using DMEM containing 10 vol.% FBS and 1 vol.% antibiotic antimycotic solution. The cultures were incubated under saturated humid conditions at 37°C with 5% CO_2_. The medium was changed every day until reaching approximately 70% to 80% confluency. Mouse colon carcinoma cell line (CT-26) was obtained from Bioresource Collection and Research Centre (BCRC, Hsinchu, Taiwan). The controlled cell (CT-26-Ctrl) was cotransfected with pLKO-AS3W-neo plasmid and packaging plasmid and infected with virus in order to grow up the tumor cell. After antibiotic selection for 2 weeks, the CT-26-Ctrl was obtained. CT-26 cells were cultured using DMEM containing 10 vol.% FBS and 1 vol.% antibiotic antimycotic solution. The cultures were incubated under saturated humid conditions at 37°C with 5% CO_2_. The medium was changed every day until reaching approximately 70% to 80% confluency.

### MTT cytotoxicity analysis

Cell growth and cytotoxicity were determined using MTT assay. In 24-well plates, 5,000 cells were cultured in each well. These plates were divided into several groups and incubated under saturated humid conditions at 37°C and 5% CO_2_. After 24 h of incubation, the medium was replenished. Among these plates, some plates were added with sample-containing medium. In addition, the negative control contained only medium, while the positive control was medium containing 5% DMSO. After culturing for 1, 3, and 5 days, 0.1 mL of MTT solution and 0.9 mL of medium were added to each well. After incubating for another 3 h at 37°C, the medium was withdrawn and replaced with 500 μL DMSO and allowed to stand about 15 min for complete reaction. Furthermore, the plates were shaken, and the readings were taken at 570 nm using an ELISA reader (Sunrise, Tecan, Männedorf, Switzerland). Proliferation on the first, third and fifth days was calculated as follows [29]:

(2) $$\mathrm{Proliferation}\left(\%\right)=A_{c}/A_{0}\times 100\%$$

where *A*_c_ is the absorbance of each system in each day and *A*_0_ is the absorbance of the control on day 0.

### *In vitro* evaluation of chemotherapy and hyperthermia studies

*In vitro* evaluation of chemotherapy and hyperthermia was conducted using drug carriers against CT-26 cells with HFMF exposure. Briefly, CT-26 cells were incubated with liposomes, doxorubicin liposomes, magnetic liposomes, or DOX-loaded magnetic liposomes (DOX concentration: 1 μM) in a 15-mL polypropylene tube which is positioned in the center of a copper coil in the HFMF system. The cells were exposed to the HFMF for 10 min. After the treatment, the cells were washed and seeded in a 96-well plate at a density of 10,000 cells per well and were incubated for another 24, 48, and 72 h. Furthermore, the MTT assay was conducted as aforementioned method and was taken at 570 nm using an ELISA reader (Sunrise, Tecan).

## Results and discussion

**Figure 1:**
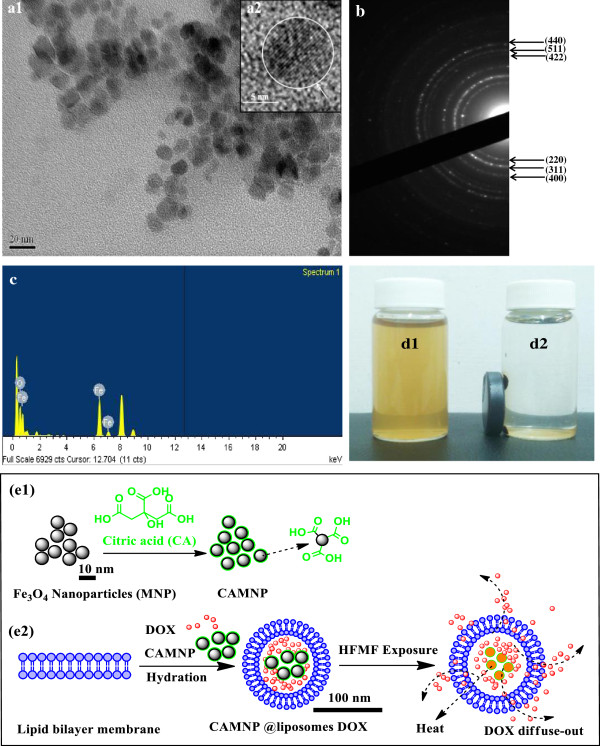
**TEM images, diffraction patterns, EDS, photographs, and diagram.** TEM image of citric acid-coated magnetic nanoparticles (CAMNP) **(a1)** and high-resolution TEM image showing the crystalline condition of iron oxide in CAMNP **(a2)**. Diffraction pattern **(b)** and EDS **(c)** of CAMNP. Photographs of CAMNP in the aqueous system, before **(d1)** and after **(d2)** the exposure of magnet. Diagram showing the proposed synthesis of surface modification MNP using CA **(e1)** and the preparation of DOX-loaded magnetic liposomes and its interaction with HFMF **(e2)**.

Figure 1a1 shows the TEM image of CAMNP which showed a spherical morphology of nanoparticles in the range of 10 nm. Under high-resolution TEM (insert Figure 1a2), CAMNP exhibited a crystalline structure. Figure 1b shows a selected area diffraction pattern of the CAMNP which showed six planes, namely, [220], [311], [400], [422], [511], and [440], indicating the presence of Fe_3_O_4_ nanoparticles. The citric acid coating might be form as a thin layer coating on the surface of MNP hence did not change any structure and crystallinity properties of magnetite nanoparticles. Furthermore, EDS analysis shows the prepared CAMNP contained the elements of Fe and O (Figure 1c). Figure 1d1 shows the CAMNP could disperse properly in the aqueous solution and exhibit the interaction with the magnetic exposure (Figure 1d2). This characteristic was generated due to the presence of carboxylic moiety on the surface of MNP (Figure 1e1). Because of their amphiphatic nature of liposomes, doxorubicin (DOX) and CAMNP are expected to be readily incorporated into hydrophilic moiety of liposomes (Figure 1e2).

**Figure 2:**
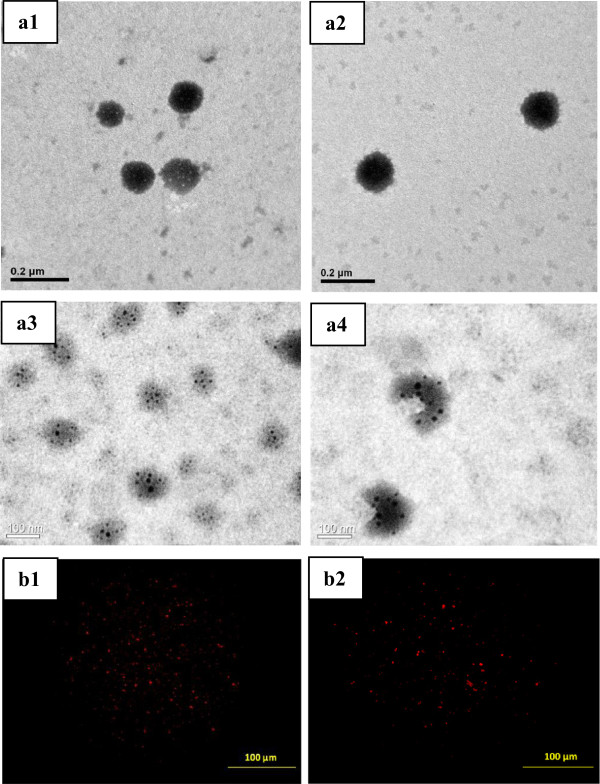
**TEM images and fluorescence microscope images.** TEM images of liposomes **(a1)**, doxorubicin liposomes **(a2)**, DOX-loaded magnetic liposomes **(a3)**, and DOX-loaded magnetic liposomes after HFMF treatment **(a4)**. Fluorescence microscope images of doxorubicin liposomes **(b1)** and DOX-loaded magnetic liposomes **(b2)**.

The morphology of liposomes, doxorubicin liposomes, and DOX-loaded magnetic liposomes was observed in TEM images with the particle size of 100, 117, and 130 nm, respectively (Figure 2a1,a3). Figure 2b1,b2 shows the fluorescence images of doxorubicin liposomes and DOX-loaded magnetic liposomes, respectively. The DOX itself carries the fluorescence properties [11] that could be explored to detect the presence of DOX in the liposomes. Liposomes without DOX did not present any fluorescence signal (not shown); meanwhile doxorubicin liposomes and DOX-loaded magnetic liposomes present the red signal which was generated from DOX. Moreover, this revealed the incorporation of DOX in both of doxorubicin liposomes and DOX-loaded magnetic liposomes.

**Figure 3:**
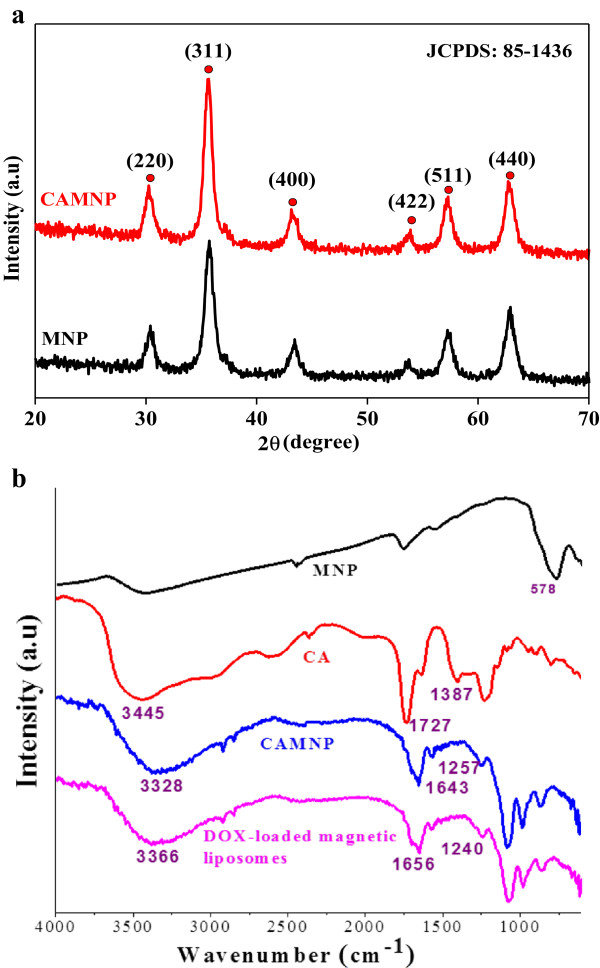
**XRD and FTIR spectra.** XRD **(a)** of pristine MNP and CAMNP. FTIR spectra **(b)** of MNP, CA, CAMNP, and DOX-loaded magnetic liposomes.

The synthesized pristine MNP and CAMNP showed X-ray diffraction pattern of multiple peaks within the 2*θ* range of 20° to 70° (Figure 3a). Six diffractions peaks at 2*θ* = 30.1°, 35.6°, 43.3°, 53.5°, 57.2°, and 62.9° were the characteristic peaks of the crystal plane which was appropriated with the standard diffraction spectrum (JCPDS: 85-1436) indicating the formation of magnetite nanoparticles [28]. The diffraction data were also used to measure the primary particle size according to Scherrer analysis for diffraction peak widths of (311) peak. The crystallite size was determined from the X-ray line broadening using Scherrer's formula *D* = 0.9*λ*/*β* cos *θ*, where *D* is the average of crystallite size, *λ* is the X-ray wavelength used, *β* is the angular line width of half maximum intensity, and *θ* is the Bragg's angle in degrees. Based on this method, the primary crystallite size of magnetite nanoparticle was estimated about 9 nm. FTIR spectrum (Figure 3b) was conducted to observe the major functional groups and molecular interaction between the components in the drug carriers. For the MNP, the characteristic absorption peaks at 578 cm^−1^[34]. The characteristic peaks of CA were shifted due to the interaction between CA and MNP nanoparticles [14,17,33]. The peaks at 3,445 cm^−1^ (hydroxyl group), 1,727 cm^−1^ (symmetric vibrational of C = O from –COOH group), 1,387 cm^−1^ (asymmetric C-O stretching from -COOH group) were shifted to 3,328, 1,643, and 1,257 cm^−1^, respectively. This observation might be attributed to the binding of CA onto the surface of MNP via chemisorption of carboxylate group [17,33]. The peak around 2,901 cm^−1^ is corresponding to the C-H stretching peaks from CH_2_ groups in CA molecules [14]. Furthermore, these results confirmed the presence of CA on the surface of MNP. In addition, the peaks corresponding to phospholipid were observed at 1,240 and 1,656 cm^−1^ for peaks of phospholipids and C = O stretching, respectively [25]. These provide the information about the liposomes layer which encapsulated magnetic nanoparticles.

**Figure 4:**
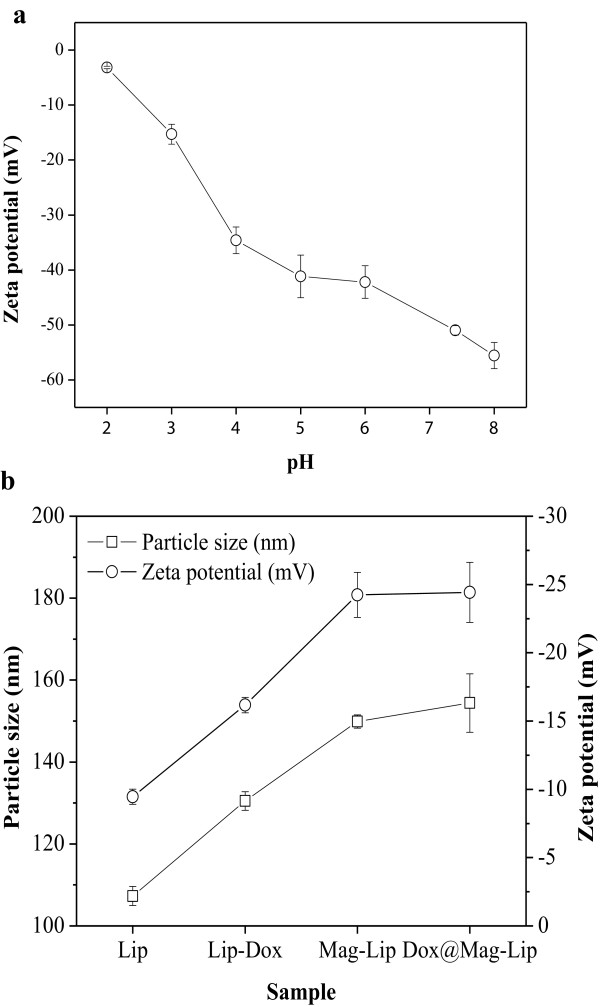
**Particle size and zeta potential measurements.** Zeta potential of CAMNP against pH values **(a)**. Particle size and zeta potential of liposomes (Lip), doxorubicin liposomes (Lip-Dox), magnetic liposomes (Mag-Lip), and DOX-loaded magnetic liposomes (Dox@Mag-Lip) **(b)**.

Figure 4a shows the relation between mean zeta potential values against pH values. High zeta potential value of CAMNP provides electrostatic and steric stabilization in the wide range of pH from the lower pH value (pH approximately 4) until physiological pH (pH approximately 7.4) which will increase the broad application of these nanoparticles for tumor or cancer treatment (lower pH), but on the other side still stable in the physiological pH condition. This result is in accordance with the characteristics previously reported [35,36]. Figure 4b (circle) shows the zeta potential value of each components in the drug carriers. Zeta potential value of pristine Fe_3_O_4_ is −40 mV. This result might be due to a number of hydroxyl groups (Fe-OH) [16]. Surface modification of MNP using CA increased the zeta potential value to −50 mV at pH 7.4. These might be due to CA adsorbed on the surface of the MNP by coordinating via one or two of the carboxylate functionalities, depending on steric necessity and the curvature of the surface. This leaves at least one carboxylic acid group exposed to the solvent, and this group should be responsible for making the surface charged and hydrophilic [17]. DLS revealed that the particle size of liposomes, doxorubicin liposomes, magnetic liposomes, and DOX-loaded magnetic liposomes was 107, 130, 149, and 154 nm, respectively (Figure 4b, square sign). This appropriate size was necessary for a drug delivery system to escape from reticuloendothelial system (RES) system [13,14,37]. This is in accordance with the characteristic previously reported [38,39]. Incorporation of CAMNP into the liposomes changes zeta potential values into −24 mV. These further prove the encapsulation of CAMNP by liposomes. In addition, negative surface charges of drug carriers may enhance the particle circulating time with avoiding of the opsonization process, the process related to the promoting of particle recognition by the cells due to the interaction between the protein entity-adsorbed particles with specific plasma membrane receptors on monocytes and various subsets of tissue macrophages [37].

**Figure 5:**
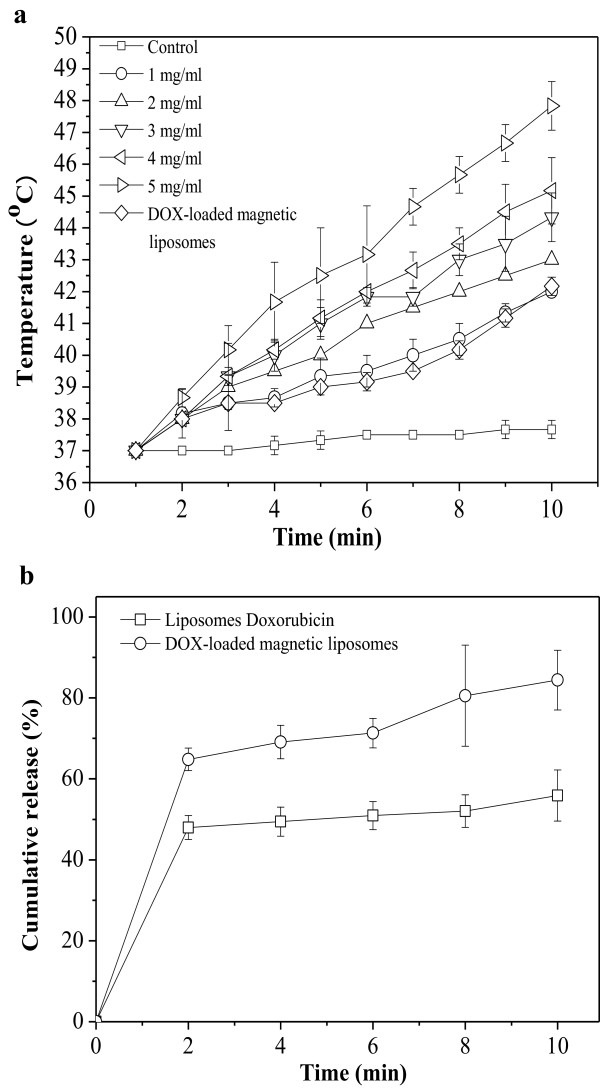
**Heating ability and cumulative release.** Heating ability of CAMNP and DOX-loaded magnetic liposomes **(a)**. Cumulative release (%) of DOX from doxorubicin liposomes and DOX-loaded magnetic liposomes with HFMF exposure **(b)**.

Figure 5a shows the results of inductive heating ability of various formulations of CAMNP and DOX-loaded magnetic liposomes. Inductive heating is thermal energy induced from the hysteresis loss of ferrites and dependent on the type of remagnetization process in the HFMF [28]. Control sample (double-distilled water) did not heat up significantly under the exposure of HFMF, and the temperature remained around 37°C. Meanwhile, DOX-loaded magnetic liposomes could reach the hyperthermia temperature (approximately 42°C) after the exposure of HFMF around 10 min. These results confirmed that the temperature increased in the DOX-loaded magnetic liposomes closely related to the incorporation of magnetic nanoparticles (CAMNP) in the liposome compartment. Based on the fluorescence spectrophotometer, drug loading of DOX was 221 and 155 ng/μl in the pristine liposomes and magnetic liposomes, respectively. The encapsulation efficiency (EE) of DOX was 22.1% for the pristine liposomes and 15.5% for magnetic liposomes. *In vitro* drug release test was conducted in order to investigate the release behavior of DOX from the drug carriers As shown in Figure 5b, the HFMF exposure generate the bursting-like release profile of DOX in DOX-loaded magnetic liposomes, about more than 80% release of DOX; meanwhile, there is no significant burst release shown by DOX encapsulated in the liposomes which is just 40% release of DOX. These phenomena might be related to the incorporation of CAMNP in the magnetic liposomes which further generate heat and initiate DOX release simultaneously. The heating induced by the exposure of HFMF might affect the structural disorder (collapse) of lipids which further generates the drug release, as shown in Figure 2a4.

**Figure 6:**
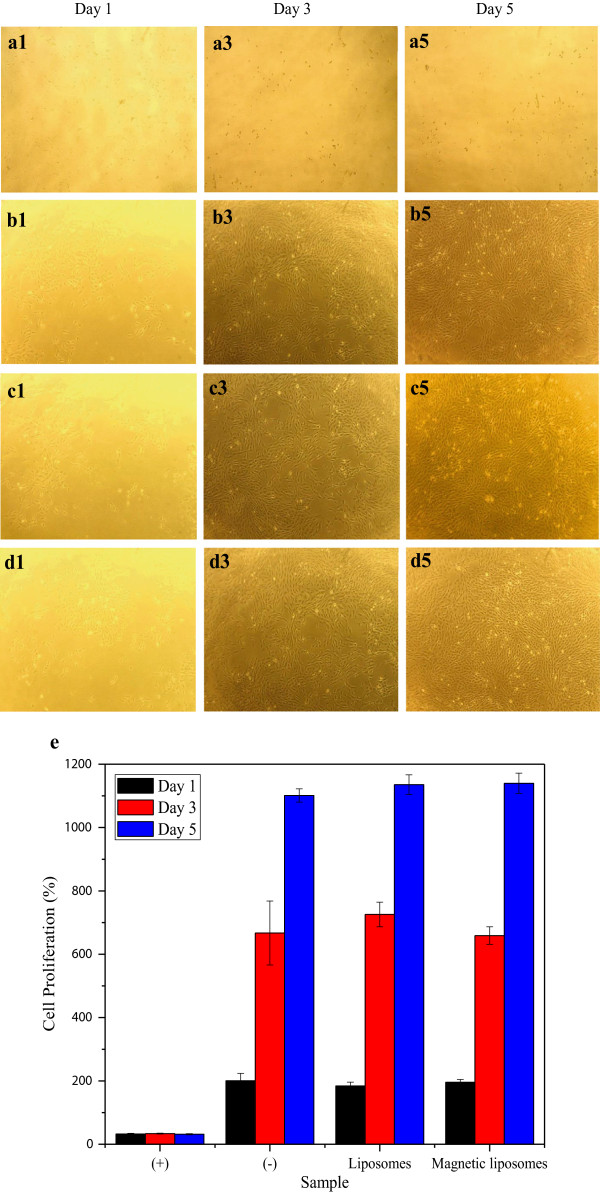
**MTT assays and cytotoxicity studies against L-929 cells.** Optical microscopy images of L-929 after incubation with positive control **(a1-a5)**, negative control **(b1-b5)**, liposomes **(c1-c5)**, and magnetic liposomes **(d1-d5)** during 1, 3, and 5 days of incubation. Cell proliferation (%) of L-929 cells during 1, 3, and 5 days of incubation **(e)**.

MTT assay was conducted to determine the cellular cytotoxicity of liposomes and magnetic liposomes using L-929 cells, as the mammalian cell model. Figure 6a shows the L-929 cells treated with cytotoxic compound (DMSO) as a positive control which resulted in the death of the cells. Moreover, as shown in Figure 6b,c,d, the morphology of the treated cells were similar with the cells in the negative group control (treated only with medium). The morphological observation using optical microscope confirmed that no toxicity effects were found compared to the negative control, indicating that the drug carrier component exhibited less influence on the growing of the cells as the cells remained viable and continued to proliferate. Figure 6e shows the cell proliferation based on the MTT assay. Based on the cell proliferation of L-929 fibroblasts cells, we found that the cells grow during the day of incubation. Liposomes and magnetic liposomes exhibited a good proliferation probably due to the biocompatibility of the liposome system with the cell compartment. Previous study revealed that the encapsulation of surface-modified magnetic nanoparticles into the liposome compartment totally resolved the problems related to the physical behavior of charged surface nanoparticles which tend to react with the serum proteins which might be present on the cell culture medium [40]. Eventually, these results confirmed that drug carriers exhibited no cytotoxicity against L-929 cells, suggesting good biocompatibility. This is in accordance with similar characteristic previously reported [41,42].

**Figure 7:**
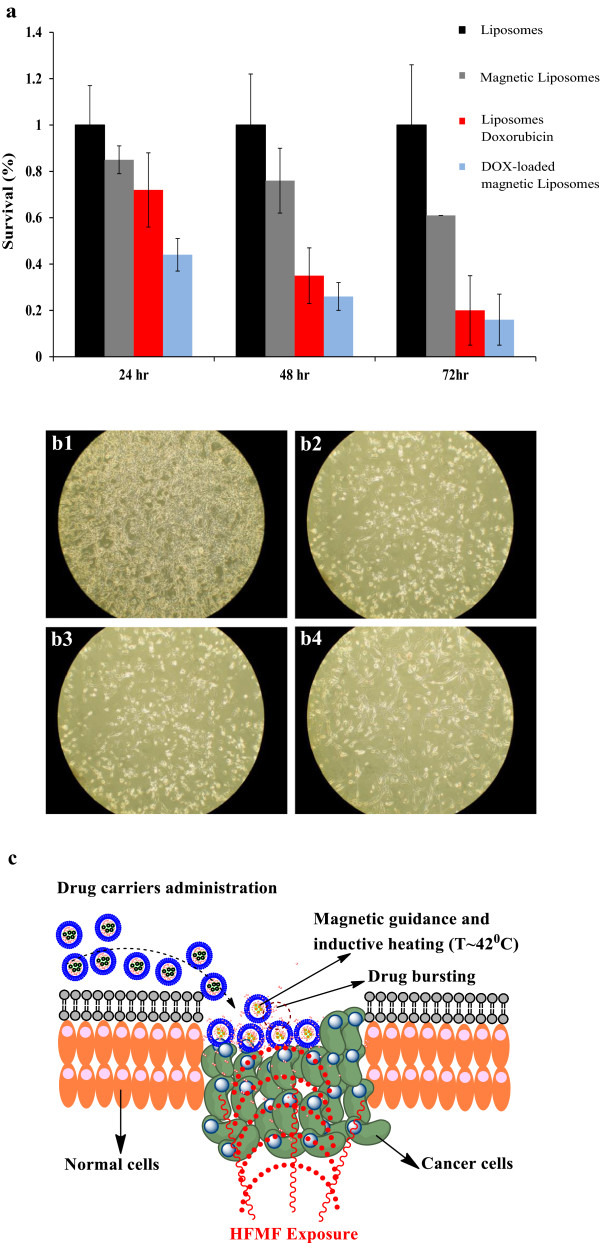
**MTT assays and cytotoxicity studies against CT-26 cells.** After HFMF exposure with liposomes, magnetic liposomes, doxorubicin liposomes, and DOX-loaded magnetic liposomes **(a)**. Optical microscope images of CT-26 cells after treated with liposomes **(b1)**, magnetic liposomes **(b2)**, doxorubicin liposomes, **(b3)**, and DOX-loaded magnetic liposomes **(b4)**. Proposed mechanism of HFMF treatments in combination with DOX-loaded magnetic liposomes **(c)**.

Figure 7a shows the effect of the *in vitro* hyperthermia treatment with various samples (contained 1 μM of DOX) against CT-26 cells, and Figure 7b shows the optical microscope image of CT-26 cancer cells after treated with various formulations. After HFMF treatment, blank liposomes exhibited no effect to the apoptosis of cancer cells. Liposomes contained CAMNP exhibited a cell cytotoxicity effect which resulted in 15% cancer cell death (after 1 day of incubation). These killing effects might be because of the hyperthermia effects through the absorption of heat by CAMNP. On the other hand, the liposomes that contain DOX also exhibited the apoptosis effect to the cancer cells after treatment using HFMF. This formulation killed 38% (after 1 day of incubation) of cancer cells during the treatment of HFMF. These phenomena might be because of the structure disruption of the liposomes when HFMF was applied to them and generate the releasing of DOX into the cancer site. These results directly proved the chemotherapeutic effects of DOX to the cancer cells. DOX is known to interact with DNA by intercalation and inhibition of DNA biosynthesis [43]. Interestingly, the formulation of DOX-loaded magnetic liposomes exhibited the highest degree of cytotoxicity to the CT-26 cancer cells which further influence cell apoptosis (56% of cancer cells were killed, after 1 day of incubation). In this case, CAMNP will absorb and generate heat when HFMF is applied to them. This heating absorption is useful for driving force for DOX release and also provide the heating (thermotherapy) effect to the cancer cells [16]. DOX encapsulated in magnetic liposomal system are expected to be readily delivered to the tumor cells due to the nonphysical interaction between HFMF and CAMNP. Hence, the combination of the tri-components with HFMF treatment will generate multifunctional effects to kill the cancer cells (Figure 7c). In biological perspective, hyperthermia might kill the cells by irreversibly damaging different cellular structures like cell membrane and cytoskeleton and causing damage to enzyme complexes required for DNA synthesis and repair. Hyperthermia irreversibly damages the actin and tubulin structures of cells which would lead to apoptosis [14]. Hyperthermia also could increase DOX absorption due to the addition of blood flow in the tumor region and decrease the tumor vessel regeneration for the reduction of synthesis and secretion of VEGF [44]. Eventually, the integrating therapy system of hyperthermia could increase the cytotoxic effects against cancer cells. These results suggest the promising application of this system for cancer treatment.

## Conclusions

Formulation of liposomes containing doxorubicin and magnetic nanoparticles has been developed as a part in the integrating treatment of chemotherapy and hyperthermia with the introduction of high-frequency magnetic field system. Cytotoxicity screening of blank drug carriers against mammalian cells, L-929 fibroblast cells, showed that these drug carriers exhibited no cytotoxicity against cellular system which further emphasized these drug carriers as a potential candidate for biocompatible materials. However, the combination between DOX and magnetic liposomes (drug concentration 1 μM) and high-frequency magnetic field treatment synergistically would increase the cytotoxicity effects to kill colorectal cancer cells. Eventually, these results confirmed the potential application of this drug carrier for cancer treatment through combination between chemotherapy (drug-controlled release) and hyperthermia (inductive heating).

## Competing interests

The authors declare that they have no competing interests.

## Authors’ contributions

AH, LYH, TYL, and MCY had conceived and designed the experiments. AH, SCT, CYK, LYH, TYC, and HMZ performed the experiments. AH, SCT, LYH, CYK, CYY, TYC, HMZ, WNL, and CHL contributed ideas and material analyses. AH, TYL, and MCY wrote the manuscript. All authors read and approved the final manuscript.

## Authors’ information

AH is a PhD student at National Taiwan University of Science and Technology. LYH is a postdoctoral fellow at National Taiwan University of Science and Technology. MCY holds a professor position at National Taiwan University of Science and Technology. TYL holds an assistant professor position at Ming Chi University of Technology. SCT is a researcher at National Yang-Ming University. CYY holds an assistant professor position at National Yang-Ming University. CYK is a PhD student at National Taiwan University. TYC and HMZ are undergraduate students at Ming Chi University of Technology. WNL is a postdoctoral fellow at National Yang-Ming University. CHL holds a professor position at National Yang-Ming University.
